# 1,2,3,4-Tetrahydro-1,4,5,8-tetraazaanthracene revisited: properties and structural evidence of aromaticity loss

**DOI:** 10.3762/bjoc.15.203

**Published:** 2019-08-28

**Authors:** Arnault Heynderickx, Sébastien Nénon, Olivier Siri, Vladimir Lokshin, Vladimir Khodorkovsky

**Affiliations:** 1 Aix Marseille Université, CNRS CINaM UMR 7325 Campus de Luminy, case 913, 13288, Marseille, France

**Keywords:** aromaticity, density functional calculations, heterocycles, hydrogen bonds, X-ray structures

## Abstract

The synthesis and properties of 1,2,3,4-tetrahydro-1,4,5,8-tetraazaanthracene (THTAA) – a heterocycle involving both >N–H donating and =N– accepting moieties – have been reinvestigated. Unlike previously reported, THTAA is a thermally stable compound that can be re-sublimed at 300–310 °C without decomposition. Controlled introduction of substituents at the nitrogen atoms of THTAA led to variation of its electron donor/acceptor capability that allowed fine-tuning the absorption properties. The propensity of these compounds and a number of its derivatives to form infinite chains involving >N–H···N= and >N–H···Hal^−^···N^+^ atoms is demonstrated by X-ray structure analysis. The DFT level optimized and experimental geometry of THTAA and its derivatives show considerable loss of aromaticity within the quinoxaline moiety.

## Introduction

**Figure 1:**
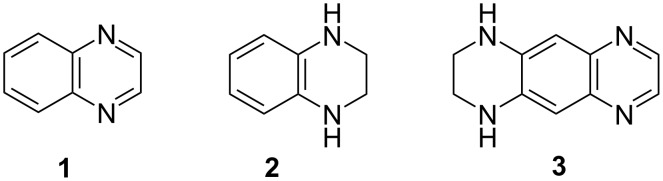
Quinoxaline derivatives **1**–**3**.

Quinoxaline derivatives **1** – also called benzopyrazines – are among the most studied heterocycles owing to their remarkable implications in many areas of chemistry, biology, medicine and agriculture. The literature on these derivatives as well as their saturated analogs **2**, covering their chemistry and applications up to 2012, has been thoroughly reviewed, in particular, in three volumes of Chemistry of Heterocyclic Compounds (Editors A. Weisssberger et al.) [1–3] and a recent book [4]. During the past years azaacenes attracted interest as potential components of light emitting diodes and electronic devices [5–7]. Surprisingly, little is known about 1,2,3,4-tetrahydro-1,4,5,8-tetraazaanthracene (1,2,3,4-tetrahydropyrazino[2,3-*g*]quinoxaline, THTAA, **3**) combining the structural elements of both **1** and **2** (Figure 1), although its isolation and preparative scale synthesis has been described more than 50 years ago [8–9] and modified later [10]. A strongly fluorescent compound, **3**, has been isolated from the reaction of ethylenediamine with noradrenaline, 2-methylnoradrenaline, adrenolone, 3,4-dihydroxymandelic acid or catechol [8].

**Scheme 1:**
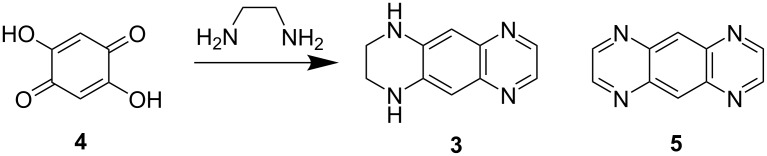
Synthesis of THTTA (**3**).

The reaction with 2,5-dihydroxy-*p*-benzoquinone (**4**) under a stream of air affording **3** in 50% yield was proposed as the most convenient method of preparation (Scheme 1) [8]. This compound was described as yellow plates (from ethanol), which decomposed at about 300 °C giving a small amount of almost colorless crystalline sublimate identified as 1,4,5,8-tetraazaanthracene (**5**) [8]. The structure of **3** as the 1,2,3,4-tetrahydro isomer has been assigned considering only indirect evidences so that the structure corresponding to the 1,2,6,7-tetrahydro isomer cannot be excluded (no NMR data have been reported so far). A well related known example involves the correct structure of fluorindine (5,14-dihydro derivative of quinoxalino[2,3-*b*]phenazine), which has been fully established in 1987 by the ^1^H NMR spectrum splitting patterns [11], the compound assumed to be the 5,12-dihydro isomer since 1890 [12].

1,2,3,4-Tetrahydro-1,4,5,8-tetraazaanthracene (THTAA, **3**) bearing the electron-donating NH and accepting =N moieties allows further modifications at these units to achieve fine-tuning of the light absorption and redox properties. In the course of this study, we also expected that these derivatives could serve as interesting components of supramolecular structures that can assemble in a predictable way owing to the formation of hydrogen bonds, an approach known as ‘crystal engineering’ (see, for instance, [13–14] and references therein). Indeed, whereas several two-component molecular systems cocrystallizing in a special layered way involving pyrazine, quinoline and phenazine as the H-bond acceptor and hydroquinones or chloranilic acid as the H-bond donors (the O–H···N=C H-bonds) are known [15–16], the examples of the N–H···N=C bonds are less numerous and involve mostly aminopyrimidines and imidazoles [13–14]. Derivatives of **3** can be of special interest as flexible supramolecular synthons [14,17] owing to the presence of two H-donor (N–H) and two H-acceptor (N=C) sites that can lead to well organized H-bonded arrangements in the solid state. Moreover, the presence of two different types of nitrogen atoms affords the possibility to be selectively substituted at the desired position.

Here we revisited the synthesis and purification of THTAA (**3**), and report on the results of our investigation based on the quantum mechanical calculations, X-ray structures and electronic absorption spectra of **3** and a number of its derivatives.

## Results and Discussion

### Synthesis, purification and chemical modification of THTAA

Derivative **3** was prepared from 2,5-dihydroxy-*p*-benzoquinone and ethylenediamine as brownish-yellow solid according to the recommended procedure (Scheme 1) [8].

Crystallization from butyl acetate produced yellow crystals of THTAA (**3**) in about 50% yield, still slightly brownish, along with the unidentified dark brown poorly soluble solid. The crystallized samples of THTAA can be sublimed at about 300 °C at ambient pressure without decomposition affording bright yellow needles in contrast to the observation of decomposition in the original paper [5]. The formation of tetraazaanthracene (TAA, **5**) was observed upon attempts to sublime the brown byproduct about 200–210 °C affording colorless crystals. The NMR spectrum corresponded to **5** (described, sometimes, as light yellow solid [18–19]).

It is worth of noting that sublimed **5** does not fluoresce so that previously observed fluorescence [20] and yellowish color of **5** stems in fact from the admixture of **3**.

**Scheme 2:**
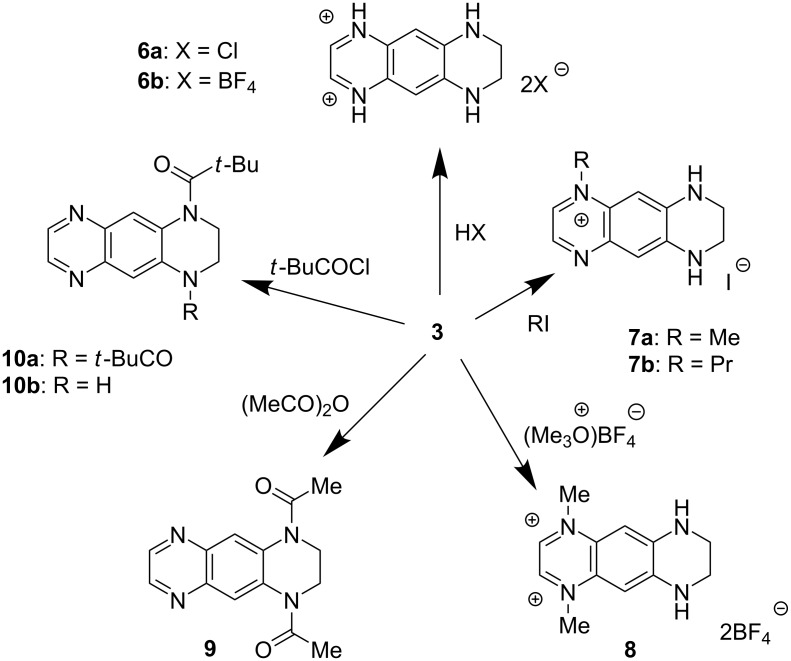
Protonation, alkylation and acylation of **3**.

We first envisaged the protonation of **3** in order to isolate the corresponding diiminium salts which should have a stronger electron-withdrawing character (compared to **3**). Acidification of **3** in ethyl acetate by an excess of HCl or HBF_4_ yielded red salts **6a** and **6b** in 85 and 78% yield, respectively (Scheme 2). The ^1^H NMR spectra of both salts are similar. We then decided to alkylate **3** in order to overcome the inherent pH-dependent behavior of salts **6**. Analogously to 4-aminopyridine [21], only the sp^2^ nitrogen atom of the quinoxaline moiety undergoes alkylation by methyl iodide or propyl iodide in MeCN for 2 days to afford **7a** and **7b** in 90 and 92% yields, respectively (Scheme 2). The dimethyl derivative **8** can be prepared using a stronger methylating agent such as trimethyloxonium tetrafluoroborate. Comparison of the ^1^H NMR spectra clearly demonstrated the formation of **8** which shows 5 signals whereas the spectrum of **7a** (or **7b**) exhibits 12 resonances. Next, we found that acylation of **3** with a large excess of acetic anhydride or pivaloyl chloride in the presence of *N*,*N*-diisopropylethylamine afforded 1,4-diacyl derivatives **9** and **10a** in high yields. Interestingly, the reaction with pivaloyl chloride in MeCN in the presence of triethylamine at 80 °C gave the monosubstituted derivative **10b** in about 48% yield even with the excess of the acylating agent. The ^1^H NMR spectra of **9** and **10a**, like in the case of derivative **8**, indicated a symmetrical structure expected for the bis-acylated products.

### Molecular structures

**Figure 2:**
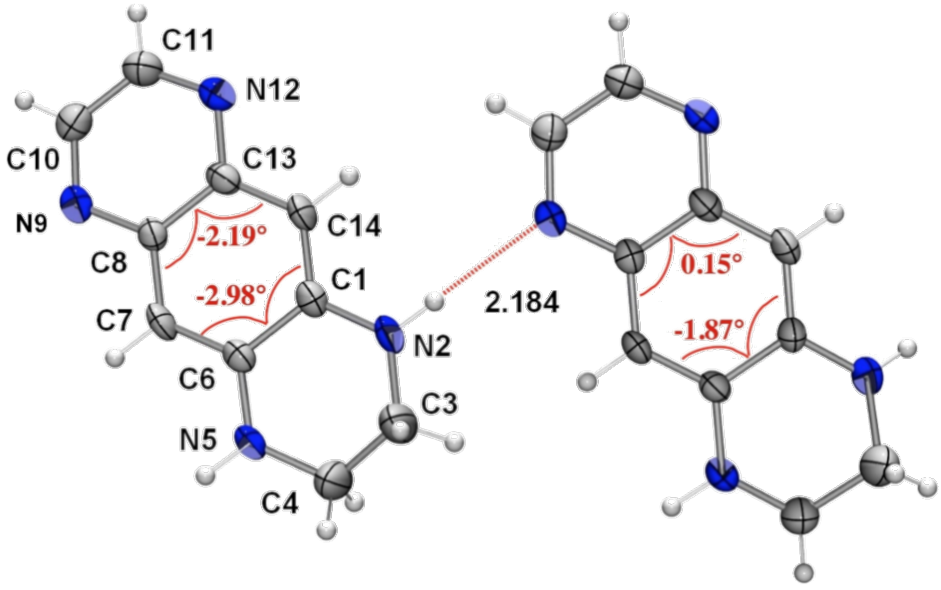
The ORTEP view of **3**. Torsion angles within the benzene ring are given in red.

The molecular structures of compounds **3**, **6a**, and **7a** have been established by an X-ray diffraction study. The molecular geometry is strongly affected by the presence of networks of intramolecular short distances: =N···H–N< (derivatives **3** and **7a**) and N–H···Cl (**6a**). The experimental (averaged) and calculated selected bond lengths are listed in Table 1. There are two independent molecules of **3** in the cell, bound with the short =N···H–N< hydrogen bond of 2.184 Å (Figure 2). Examination of the bond distances within the N2–C1–C14–C13–N12, N5–C6–C7–C8–N9 atom chains and the respective bond lengths of the second independent molecule of **3** reveals a contribution of the intramolecular charge transfer from the electron donating ethylenediamino moiety (D) toward the electron accepting ethylenediimino (A) moiety as shown by the narrow range of the C–N averaged distances from 1.363 to 1.367 Å (Table 1). The C1–C2 and C4–C5 distances vary between 1.447 and 1.421 Å, respectively, indicating poor conjugation through these bonds, as a result of a partial loss of the aromaticity of the central benzene ring. This feature is confirmed also by the deviation of the benzene ring planarity as shown in Figure 1: the torsion angles C7–C6–C1–C14 and C7–C8–C13–C14 exceed 2 degrees. The planarity distortion cannot be attributed solely to the intramolecular interaction effects, as our calculations [22] using the B3LYP/6-311+G(2d,p)//B3LYP/6-311+G(2d,p) model chemistry also reproduce the distortion, albeit to a smaller extent: 1.07 and 0.2 degrees. The frequency calculation reproduces the experimental IR spectrum reasonably well (Supporting Information File 1, Figure S1).

**Table 1:** Experimental and calculated selected bond lengths (Å).

Compound	Bond	Experimental^a^	Calculated

**3**	C1–N2	1.367	1.391
	C1–C14	1.371	1.376
	C13–C14	1.405	1.411
	C13–N12	1.363	1.355
	C11–N12	1.327	1.317
	C1–C6	1.445	1.441
	C8–C13	1.425	1.430
**6a**	C1–N2	1.335	1.329
	C1–C14	1.384	1.398
	C13–C14	1.389	1.381
	C13–N12	1.345	1.357
	C11–N12	1.370	1.361
	C1–C6	1.469	1.477
	C8–C13	1.441	1.459
**7a**	C1–N2	1.342	1.352
	C1–C6	1.378	1.393
	C5–C6	1.403	1.393
	C5–N4	1.365	1.371
	C2–N1	1.339	1.366
	C2–C3	1.380	1.374
	C3–C4	1.388	1.409
	C4–N3	1.357	1.408
	C1–C2	1.457	1.458
	C4–C5	1.449	1.448

^a^Averaged bond lengths for **3** and **6a**.

**Figure 3:**
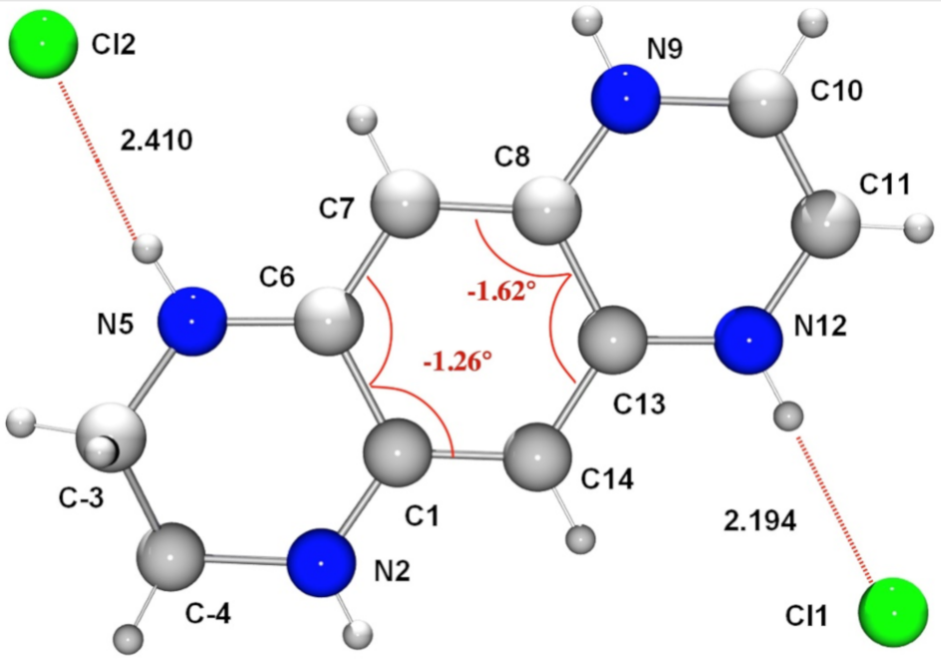
The ORTEP view of **6a**. Torsion angles within the benzene ring are given in red.

The X-ray structure determination of **6a** confirms that the protonation occurs at 5,8-positions (Figure 3). The distances between both >N–H and =N–H groups from the chlorine anions are shortened (2.410 and 2.194 Å, respectively, Figure 2). Examination of the bond distances within the N2–C1–C14–C13–N12 and N5–C6–C7–C8–N9 atom chains of **6a** reveals almost full delocalization of the positive charge along the both conjugated moieties (Table 1). The bonds of C1–C6 (1.469 Å) and C8–C13 (1.441 Å) are also elongated, indicating further loss of aromaticity within the dication. The planarity distortion is smaller than observed in **3**, judging by the torsion angles C7–C6–C1–C14 and C7–C8–C13–C14 (Figure 3). At the same time, the geometry optimized structure of **6a** exhibits much greater torsion angles of 6.23 and 2.28 degrees.

**Figure 4:**
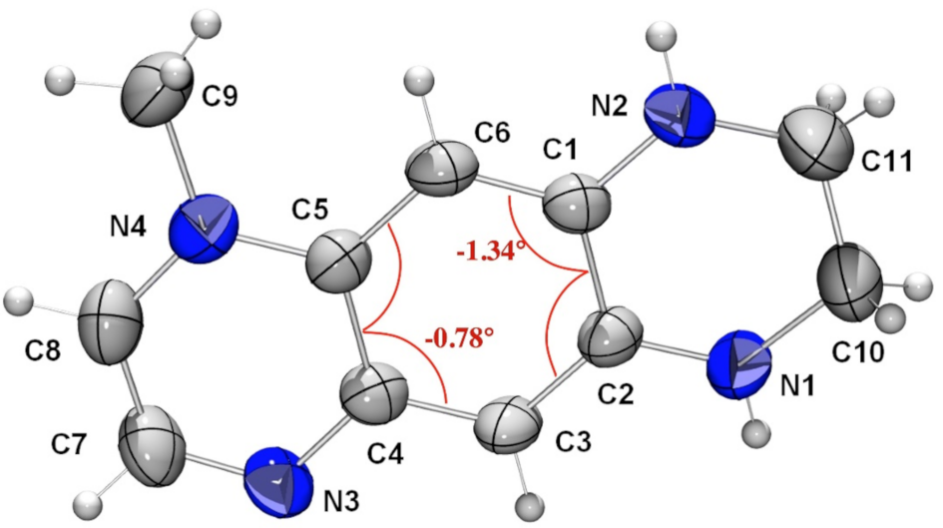
The ORTEP view of **7a** (the iodine counter anion has been omitted for clarity). Torsion angles within the benzene ring are given in red.

The X-ray structure analysis of **7a** confirms that the methylation occurs at the N4 atom to form the respective cation (Figure 4). Examination of the bond distances revealed a significant degree of the positive charge delocalization predominantly over the N2–C1–C6–C5–N4 moiety of the molecule. The torsion angles within the benzene ring are smaller than calculated (1.09° and 1.95°, respectively) and those of found in **3** and **6a**.

**Scheme 3:**
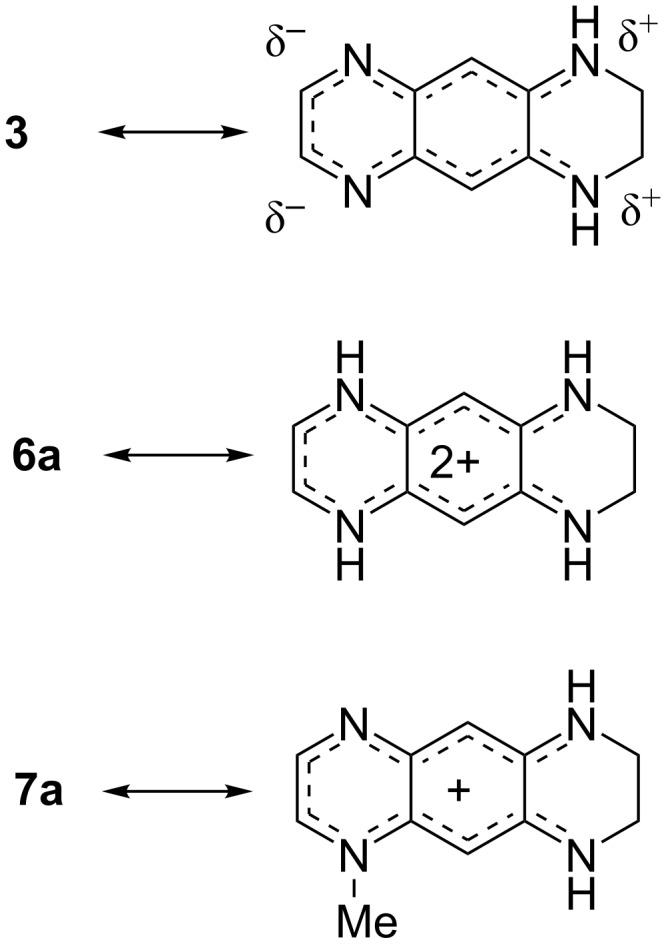
Charge transfer and delocalization within **3** and its diprotonated (**6a**) and monomethylated (**7a**) derivatives.

The above observations suggest that the degree of aromaticity of quinoxaline derivative **3** decreases compared to aromatic and planar **1** [23–24] because of the presence of the amino groups that give rise to intramolecular charge transfer. This effect is even stronger in **6a** and **7a** because of delocalization of the positive charge. The equilibrium geometries of **3**, **6a** and **7a** obtained by calculations are in good agreement with the X-ray structural data (Table 1). The geometry optimized structures confirm that the bond lengths equalization and the aromaticity loss observed in the X-ray structures stem from the intramolecular charge transfer from the electron-donating ethylenediamino moiety toward the electron-accepting ethylenediimino moiety in **3** and delocalization of the positive charges in **6a** and **7a** (Scheme 3), and are not artifacts arising from the strong intermolecular interactions within the crystals. Losing aromaticity, the benzene ring is becoming less rigid and the molecules can become more or less planar to adopt the constrains of the molecular packing. Thus, the structures of salts **6a** and **7a** resemble more those of cyanine dyes and the structure of **3** those of merocyanines.

The protonation positions of **3** are correctly predicted by the calculations. For instance, the energy difference between the dication **6** and a hypothetic dication with protonated amino groups is 51 kcal/mol in favor of **6**.

### UV–vis absorption spectra

**Figure 5:**
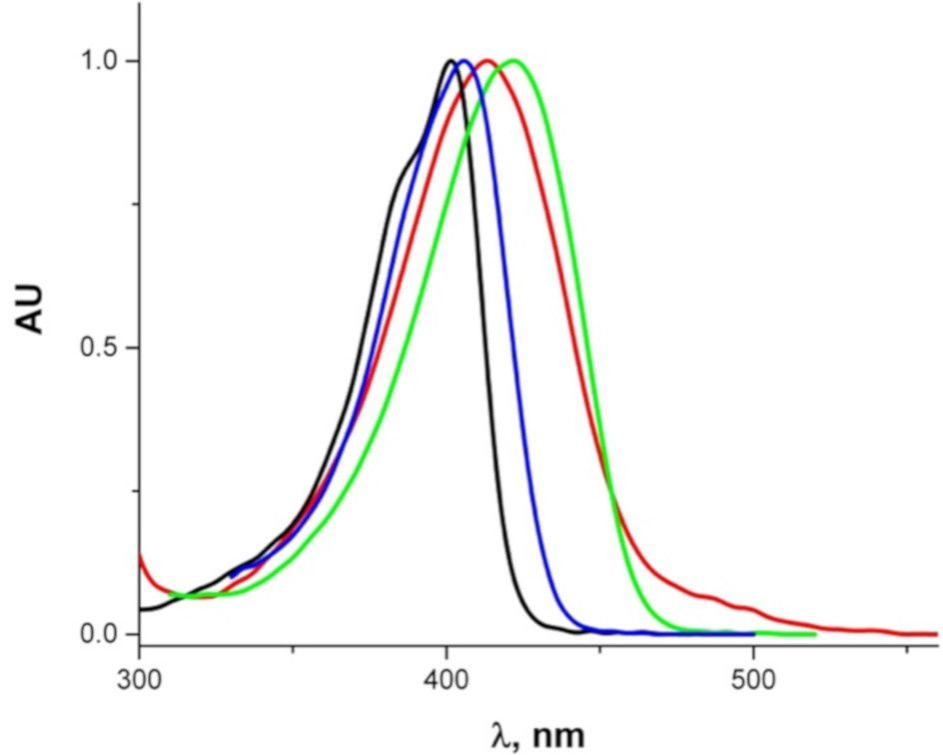
Normalized absorption spectra of **3** in: toluene (black), acetone (blue), ethanol (green), water (red).

In solution, **3** is characterized by a strong absorption band, at 402 nm (toluene), 406 nm (acetone), 413 nm (water) and 422 nm (ethanol) (ε ≈ 20,000). Whereas a 5 nm shift between the absorption maximum in toluene and acetone evidences the presence of slight positive solvatochromism, the band shapes in water and ethanol are irregular and reflect rather specific interaction with these solvents. The shoulder extending from 450 till 540 nm observed in water disappears upon addition of a base (KOH) and indicates partial protonation (Figure 5). In non-polar solvents this band shows features of weak vibronic splitting. Minimum three Pekarian functions are needed to reproduce the band shape in toluene indicating that at least three electronic transitions are involved (Supporting Information File 1, Figure S2).

**Figure 6:**
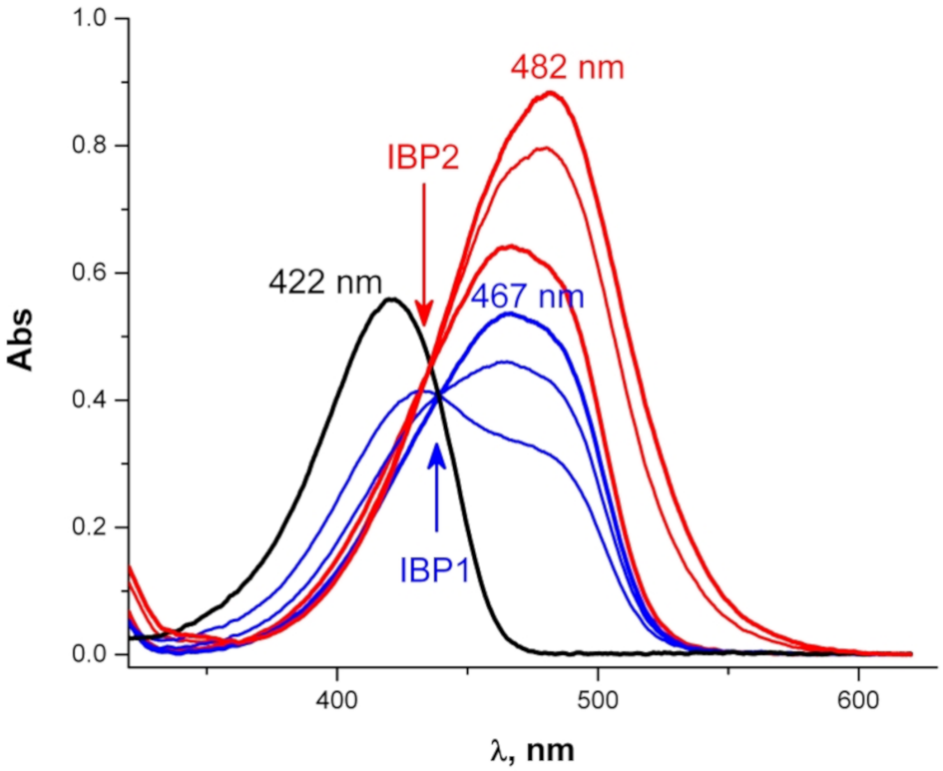
Protonation of **3** in ethanol. Two isosbestic points (IBP) are indicated by arrows.

Protonation of **3** with acids (HCl or HBF_4_ in ethanol) occurs in two separate steps (Figure 6) and the formation of the dication requires an excess of the acid. The dropwise addition of the acid to the solution of **3** gives rise to the appearance of an absorption band at 467 nm corresponding to the monoprotonated species (blue curves, IBP1). Further addition of the acid leads to the appearance of another absorption band at 482 nm corresponding to the bis-protonated species (red curves, ABP2). The protonated species of **3** are not fluorescent. Minimum two Pekarian functions are needed to reproduce the band shape of the salts **6** in ethanol indicating that at least two electronic transitions are involved (Supporting Information File 1, Figure S3).

TD DFT calculations interprets the longest wavelength absorption bands of **3** and **6** to be predominantly the charge transfer type HOMO -> LUMO transitions (Supporting Information File 1, Tables S1 and S2).

The easiness of protonation and stability of salts **6** evidenced the behavior of THTAA (**3**) as a strong base. Indeed, our DFT calculations of the proton affinity of THTAA (**3**) produced Δ*H*^298^ = 235.9 kcal/mol. For comparison, we calculated the proton affinities of quinoline: 217.0 and 4-dimethylaminopyridine: 240.1 kcal/mol at the same level of theory (the experimental values for these two derivatives are 214.4 and 235.7 kcal/mol, respectively [25]). Thus, the basicity of **3** is close to that of 4-dimethylaminopyridine.

**Figure 7:**
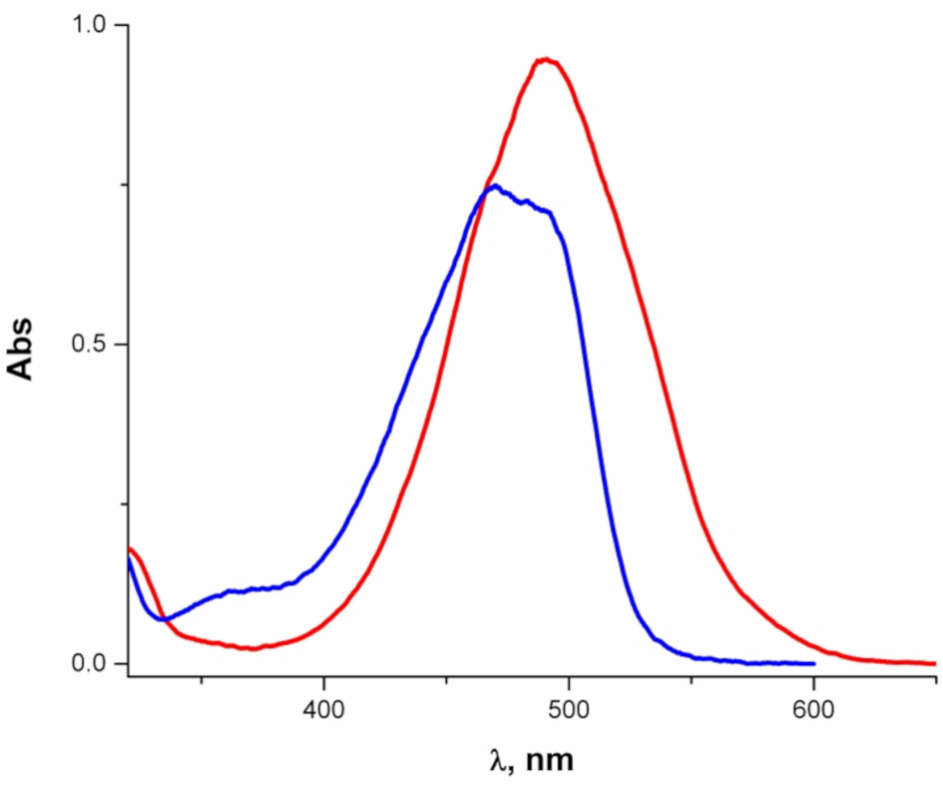
Absorption spectra of **7a** (blue) and **8** (red) in ethanol.

The absorption spectra of **7a** and **8** (Figure 7) resemble those of the mono- and diprotonated species of **3** (Figure 5), respectively.

**Figure 8:**
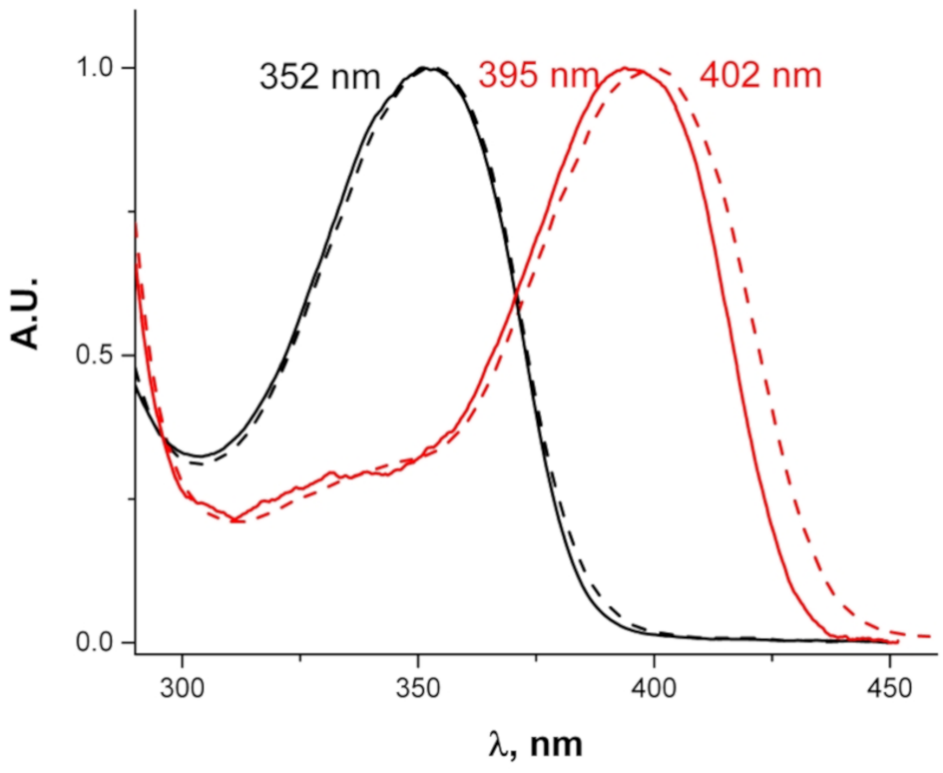
Absorption spectra of **10a** (black), **10b** (red). Solid curves: in toluene, dashed curves: in acetone.

The absorption spectra of the acylated derivatives **10a** and **10b** are shown in Figure 8. Whereas the mono-acylated derivative **10b**, which retains one electron-donating substituent (N–H), absorbs at about the same wavelength as **3** and exhibits a weak positive solvatochromic shift of about 7 nm, absorption of bis-acylated derivative **10a** is strongly blue shifted and shows no solvatochromism.

As noted in [5], both **3** and **5** undergo rapid photoreactions in solution in the presence of moisture. The photochemistry of both compounds is currently under investigation in our labs.

### Crystal structures

**Figure 9:**
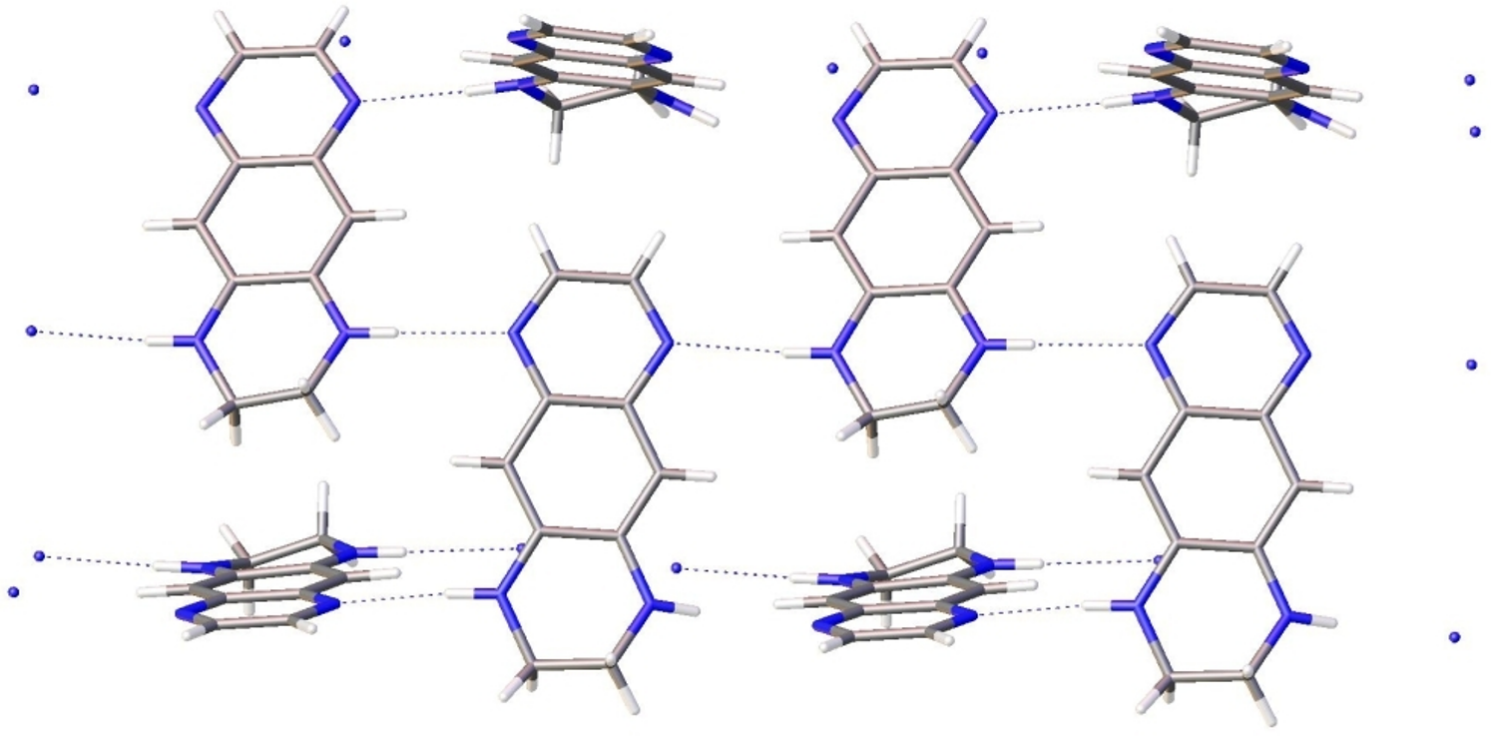
View of the supramolecular array generated by **3** in the solid state. Color coding: nitrogen, blue; carbon, grey.

Numerous short intermolecular contacts are revealed upon inspection the crystal lattice of **3**, **6a** and **7a**. There are two independent molecules of **3** in the unit cell with slightly different bond lengths. The dominant motive seen for **3** in the solid state is the formation of infinite chains based on N–H···N=C hydrogen bonds (N···N distances 3.011–3.047 Å, Figure 9).

**Figure 10:**
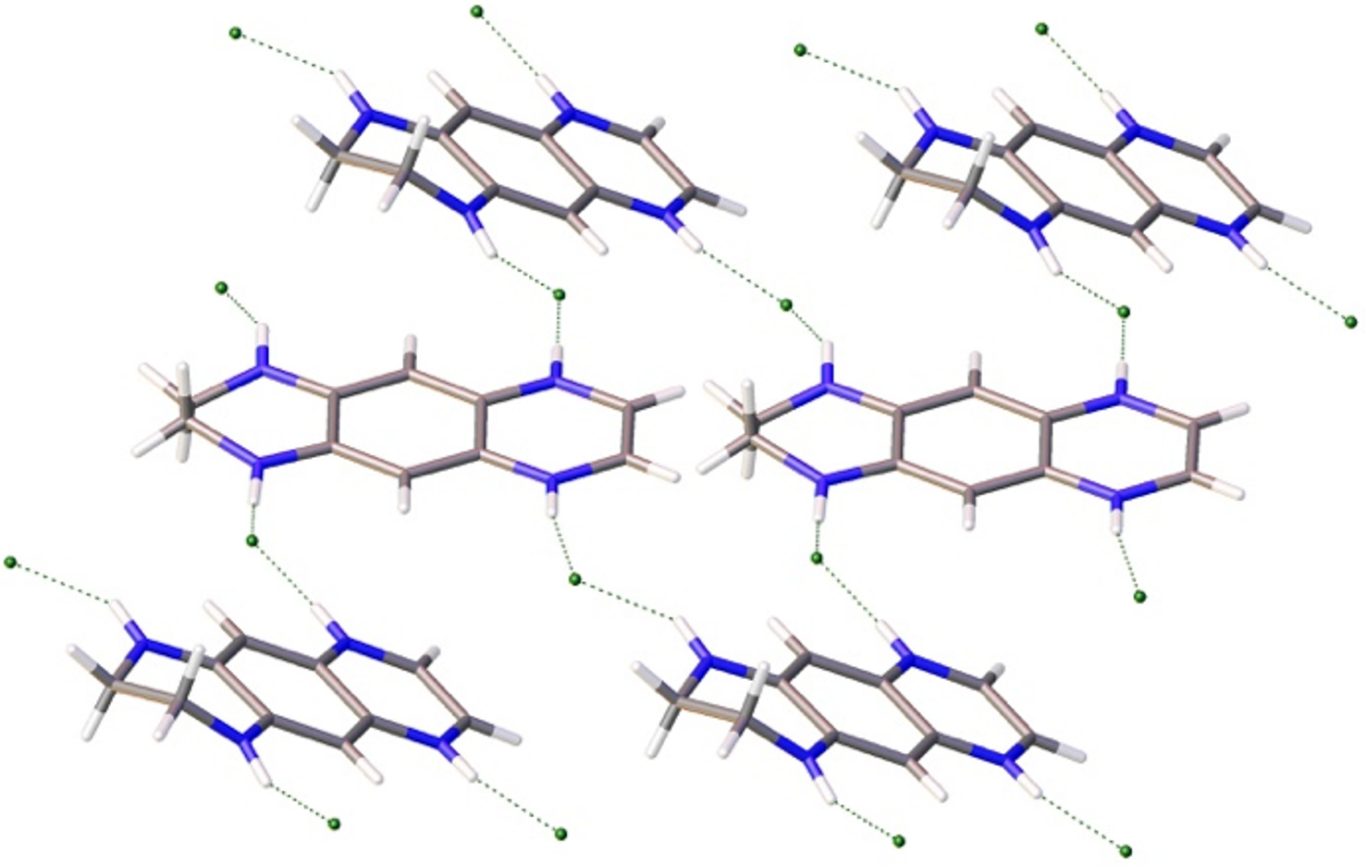
View of the supramolecular array generated by **6a** in the solid state. Color coding: nitrogen, blue; carbon, grey, chloride, green.

The crystal lattice of **6a** (Figure 10) also exhibits the presence of the infinite chains >N–H···Cl^−^···H–N^+^: the Cl^−^···N and Cl^−^···N^+^ distances being 3.184 and 3.042 Å, respectively. These interactions, as well as intramolecular charge transfer from the electron-donating ethylenediamino moiety to the electron-accepting ethylenediimino moiety give rise to a certain degree of the single and double bond lengths equalization. Thus, **6a** forms a 2D supramolecular network involving the chloride anion Cl1 as an intermolecular connector between the molecules. Each anion interacts with the two N–H protons of the associated dications (Figure 10).

**Figure 11:**
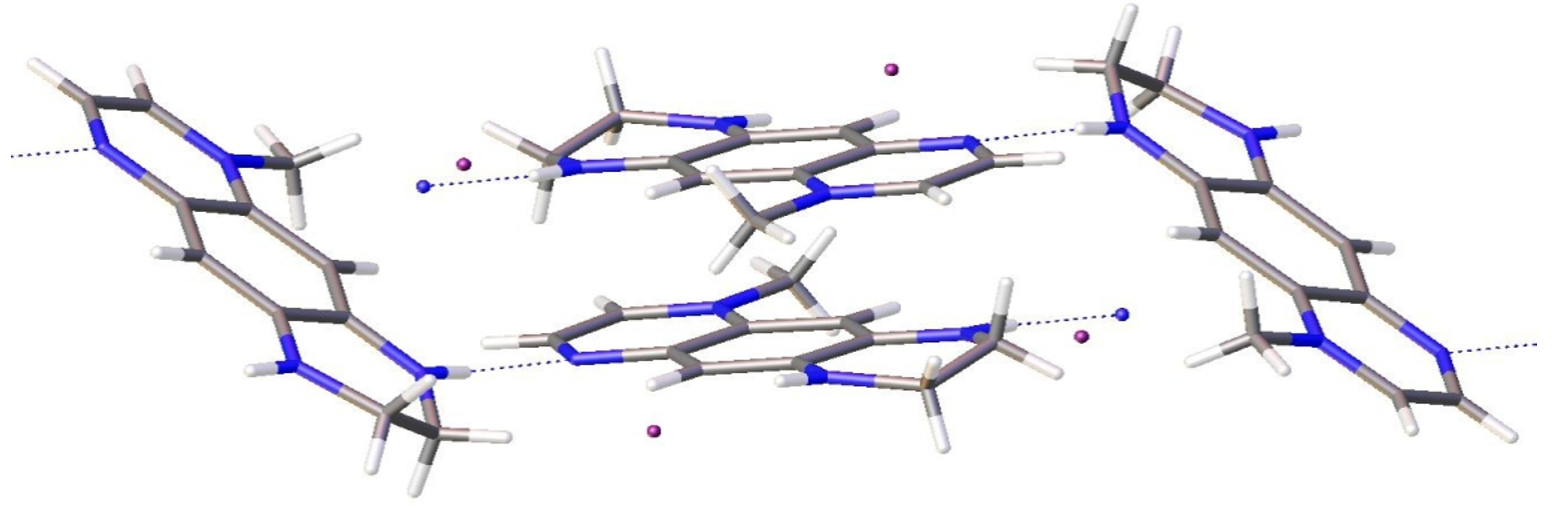
View of the supramolecular array generated by **7a** in the solid state. Color coding: nitrogen, blue; carbon, grey; iodine, violet.

In spite of the asymmetrical structure, the X-ray structure of **7a** exhibits the same trend: infinite chains of the N–H···N=C shortened distances (N(2)···N(3) distance 3.047 Å, (Figure 11). The iodine anions are not involved in the intermolecular interactions.

## Conclusion

Unlike previously described, 1,2,3,4-tetrahydro-1,4,5,8-tetraazaanthracene (**3**) is a thermally stable compound and can be sublimed at about 300 °C without decomposition. Sublimation is required for its complete purification. The crystal structures of **3** and its derivatives exhibit the formation of infinite chains binding molecules in the side-to-side manner even in the case when a substituent is present at one of the nitrogen atoms. This molecule can be easily modified at either ethylenediamino or ethylenediimino moiety and can thus serve as an attractive precursor for crystal design and creation of supramolecular assemblies. Of special interest is that the quinoxaline moiety of THTAA (**3**) and its derivatives are losing their aromaticity owing to the charge transfer from the electron-donating moiety towards the electron-accepting moieties via the benzene ring. In solution both derivatives **3** and **5** are light sensitive. Photochemical reactions of THTAA (**3**) and TAA (**5**) are currently under investigation in our labs.

## Experimental

Reagents and starting materials were used as received without further purification from Alfa Aesar and Aldrich. Analytical thin-layer chromatography (TLC) was done on Merck 60F_254_ silica gel plates from Macherey-Nagel. Detection of TLC components was accomplished using a 254/366 nm UV lamp. Column liquid chromatography was carried out on Merck silica gel 60 (70–230 mesh). Melting points were determined on a Büchi 510 apparatus in open glass capillaries and are uncorrected. The IR spectra were recorded with an Agilent 360FTIR instrument. The UV–vis absorption spectra were recorded with Ocean Optics USB 4000 and JASCO V-660 spectrophotometers. ^1^H and ^13^C NMR spectra were recorded with a JEOL JNM-ECS400 spectrometer at 399.78 and 100.53 MHz, respectively. Chemical shifts are given in ppm downfield from tetramethylsilane and coupling constants (*J*) in Hertz. Elemental analysis was made using a Thermo Finnigan EA 1112 instrument. HRMS was made at the Spectropole (http://fr-chimie.univ-amu.fr/spectropole/).

The X-ray crystallography data were collected on a Bruker-Nonius KappaCCD diffractometer with CCD detector using Mo Kα radiation, λ = 0.71073 Å. Crystallographic data (excluding structure factors) for the structures in this paper have been deposited with the Cambridge Crystallographic Data Centre as supplementary publication: **3** CCDC 1912951, **6a** CCDC 1912953, **7a** CCDC 1912952.

Quantum mechanical calculations were done with Gaussian 09 software [22] using B3LYP/6-311+G(2d,p) model chemistry. All energies include ZPE correction. 6 states were included in the TD B3LYP calculations.

**1,2,3,4-Tetrahydropyrazino[2,3-*g*]quinoxaline (3, THTAA):** THTAA was prepared as described in [8] from **4**. The brown precipitate of the crude product (50% yield) was extracted with hot butyl acetate, from which **3** precipitated as brownish yellow crystals in 50% yield. The bright yellow crystals of **3** used for recording the UV–vis absorption spectra and X-ray structure determination can be obtained only by sublimation. ^1^H NMR (DMSO-*d*_6_) δ 3.28 (s, 4H), 6.67 (br s, 2H), 6.68 (s, 2H), 8.17 (s, 2H); ^13^C NMR (DMF-*d*_7_) δ 39.84 (CH_2_), 104.88 (CH), 139.64 (CH), 140.29 (C), 140.33 (C). IR (sublimed solid sample) ν, cm^−1^: 3229–2838 (w), 1507 (s), 1340, 1315, 1218, 844, 810, 487 (see also Supporting Information File 1, Figure S1).

**1,2,3,4-Tetrahydropyrazino[2,3-*g*]quinoxaline-6,9-diium dichloride (6a):** To a suspension of 1,2,3,4-tetrahydropyrazino[2,3-*g*]quinoxaline (**3**, 500 mg, 2.68 mmol) in ethyl acetate (50 mL) was added dropwise via syringe an solution of hydrogen chloride in ethanol (1.25 M, 13.4 mmol, 10.7 mL) at 0 °C. After stirring for 2 hours at room temperature, the red precipitate was isolated by filtration in 85% yield (582 mg). ^1^H NMR (DMSO-*d*_6_) δ 3.42 (s, 4H), 6.93 (s, 2H), 8.25 (br s, 2H), 8.35 (s, 2H); anal. calcd for C_10_H_12_N_4_Cl_2_: C, 46.35; H, 4.67; N, 21.62; found: C, 46.20; H, 4.62; N, 21.35.

**1,2,3,4-Tetrahydropyrazino[2,3-*g*]quinoxaline-6,9-diium bis(tetrafluoroborate) (6b):** To a suspension of 1,2,3,4-tetrahydropyrazino[2,3-*g*]quinoxaline (**3**, 500 mg, 2.68 mmol) in ethyl acetate (50 mL) was added dropwise via syringe a commercial solution of tetrafluoroboric acid in diethyl ether (51–57%, 13.4 mmol, 1.7 mL) at 0 °C. After stirring for 2 hours at room temperature, the red precipitate was isolated by filtration in 78% yield (756 mg). ^1^H NMR (acetone-*d*_6_) δ 3.53 (s, 4H), 6.92 (s, 2H), 7.88 (br s, 2H), 8.33 (s, 2H); anal. calcd. for C_10_H_12_N_4_·2BF_4_: C, 33.19; H, 3.34; N, 15.48; found: C, 33.26; H, 3.22; N, 15.91.

**6-Methyl-1,2,3,4-tetrahydropyrazino[2,3-*g*]quinoxalinium iodide (7a):** A solution of 1,2,3,4-tetrahydropyrazino[2,3-*g*]quinoxaline (**3**), 100 mg, 0.537 mmol), iodomethane (0.33 mL, 762 mg, 5.37 mmol) in acetonitrile (7 mL) was heated in a sealed ampulla at 80 °C for 2 days. After removing the solvent in vacuo, the residue was taken up in ethyl acetate (3 mL). The obtained precipitate is then isolated by filtration and washed with ethyl acetate affording the titled product as dark red solid in 90% yield (158 mg); ^1^H NMR (DMSO-*d*_6_) δ 3.42 (m, 2H, CH_2_), 3.53 (m, 2H, CH_2_), 4.17 (s, 3H, CH_3_), 6.78 (s, 1H), 6.95 (s, 1H), 7.84 (s, 1H, NH), 8.37 (d, *J* = 3.5 Hz, 1H), 8.49 (d, *J* = 3.5 Hz, 1H), 8.79 (s, 1H, NH); anal. calcd for C_11_H_13_N_4_I·1/2H_2_O: C, 39.2; H, 4.2; N, 16.6; found: C, 39.2; H, 3.8; N, 16.6.

**6-Propyl-1,2,3,4-tetrahydropyrazino[2,3-*g*]quinoxalinium iodide (7b):** A solution of 1,2,3,4-tetrahydropyrazino[2,3-*g*]quinoxaline (**3**, 100 mg, 0.537 mmol), iodopropane (0.53 mL, 912 mg, 5.37 mmol) in acetonitrile (7 mL) was heated at 80 °C for 2 days. After removing the solvent in vacuo, the residue was taken up in ethyl acetate (3 mL). The obtained precipitate is then isolated by filtration and washed with ethyl acetate affording the product as dark red solid in 92% yield (176 mg). ^1^H NMR (DMSO-*d*_6_) δ 0.93 (t, *J* = 7.3 Hz, 3H), 1.91 (sext, *J* = 7.3 Hz, 2H), 3.40 (m, 2H, NCH_2_), 3.52 (m, 2H, NCH_2_), 4,50 (t, *J* = 7.3 Hz, 2H), 5,24 (br s, 1H, NH), 6.92 (s, 1H), 6.95 (s, 1H), 7,82 (br s, 1H), 8.36 (d, *J* = 3.6 Hz, 1H), 8.49 (d, *J* = 3.6 Hz, 1H), 8.67 (s, 1H); anal. calcd for C_13_H_17_N_4_I·H_2_O: C, 41.7; H, 5.1; N, 14.96; found: C, 42.4; H, 4.2; N, 14.1.

**6,9-Dimethyl-1,2,3,4-tetrahydropyrazino[2,3-*g*]quinoxalinium bis(tetrafluoroborate) (8):** 1,2,3,4-Tetrahydropyrazino[2,3-*g*]quinoxaline (100 mg, 0.537 mmol) and trimethyloxonium tetrafluoroborate (198 mg, 1.342 mmol, 2.5 equiv) in 12 mL of dry 1,2-dichloroethane were refluxed for 5 h. The red precipitate was filtered and washed with pentane. Recrystallisation from ethanol gave red prisms (157 mg, 75%). ^1^H NMR (DMSO-*d*_6_) δ 3.71 (s, 4H), 4.18 (s, 6H), 6.91 (s, 2H), 8.45 (s, 2H), 10.15 (s, 2H); ^13^C NMR (DMSO-d_6_) δ 38.24 (CH_2_), 44.07 (CH_3_), 93.54 (CH), 127.79 (CH), 136.85, 146.58; ^19^F NMR (DMSO-*d*_6_) δ 148; HRMS (ESIMS) *m*/*z*: [M^2+^]; calcd. for C_12_H_16_N_4_^2+^ 216.1364 108.0682; found, 108.0683.

**1,4-Diacetyl)-1,2,3,4-tetrahydropyrazino[2,3-*g*]quinoxaline (9):** 1,2,3,4-Tetrahydropyrazino[2,3-g]quinoxaline (**3**, 500 mg, 2.68 mmol) was added to 20 mL of acetic anhydride and the mixture was stirred at reflux for 48 h. After cooling, the excess of acetic anhydride was evaporated under reduced pressure. The residue was triturated with dichloromethane (20 mL) and H_2_O (20 mL). The aqueous layer was neutralized by adding solid sodium carbonate and extracted with dichloromethane (2 × 20 mL). The extracts were dried over magnesium sulfate and concentrated in vacuo. The residue was purified by column chromatography on silica gel using CH_2_Cl_2_/AcOEt/MeOH (8:2:1) as eluent, to give the titled product (696 mg, 96%) as colorless solid. Mp: 232 °C (244 °C [8]); ^1^H NMR (CDCl_3_) δ 2.37 (s, 6H), 4.05 (s, 4H), 8.06 (br s, 2H), 8.79 (s, 2H); ^13^C NMR (CDCl_3_) δ 22.91, 44.53, 122.98, 136.17, 140.59, 144.96, 169.49; anal. calcd for C_14_H_14_N_4_O_2_: C, 62.21; H, 5.22; N, 20.73; found: C, 62.31; H, 5.13; N, 20.60.

**1,4-Bis(2,2-dimethylpropanoyl)-1,2,3,4-tetrahydropyrazino[2,3-*g*]quinoxaline (10a):** To a mixture of 1,2,3,4-tetrahydropyrazino[2,3-*g*]quinoxaline (**3**, 500 mg, 2.68 mmol), *N*,*N*-diisopropylethylamine (2.33 mL, 13.42 mmol) in acetonitrile (35 mL) was added drop wise via syringe trimethylacetyl chloride (1.64 mL, 1.62 g, 13.42 mmol). The resulting mixture was stirred at 80 °C for 3 days. After cooling, the solvents were evaporated in vacuo and the residue was dissolved in dichloromethane (50 mL). The organic layer was washed with an aqueous sodium hydroxide (1 M, 2 × 5 mL), then with water (10 mL), dried over magnesium sulfate, evaporated. The crude product was purified by column chromatography on silica gel using CH_2_Cl_2_/AcOEt (4:1) to furnish the product in 87% yield (828 mg) as a yellow solid. Mp: 232 °C; ^1^H NMR (DMSO-*d*_6_) δ 1.24 (s, 18H), 4.03 (s, 4H), 8.04 (s, 2H), 8.86 (s, 2H); ^13^C NMR (DMSO-*d*_6_) δ 28.41, 40.19, 46.86, 124.16, 138.17, 139.79, 145.48, 177.59; HRMS (ESIMS) *m*/*z*: [M + H]^+^ calcd. for C_20_H_26_N_4_O_2_^+^, 355.2129; found, 355.2130.

**1-(2,2-Dimethylpropanoyl)-1,2,3,4-tetrahydropyrazino[2,3-*g*]quinoxaline (10b):** To a mixture of 1,2,3,4-tetrahydropyrazino[2,3-*g*]quinoxaline (**3**, 500 mg, 2.68 mmol), triethylamine (0.6 mL, 0.48 g, 4.56 mmol) in acetonitrile (250 mL) was added dropwise via syringe trimethylacetyl chloride (0.49 mL, 0.48 g, 4 mmol). The resulting mixture was stirred the mixture was stirred at 80 °C for 12 hours. After cooling, the solvents were evaporated in vacuo and the residue was dissolved in dichloromethane (50 mL). The organic layer was washed with a aqueous sodium hydroxide (1 M, 2 × 5 mL), then with water (10 mL), dried over magnesium sulfate, and evaporated. The crude product was purified by column chromatography on silica gel using cyclohexane/AcOEt (3:7) to furnish the titled product in 48% yield (348 mg) as a yellow solid. ^1^H NMR (CDCl_3_) δ 1.38 (s, 9H), 3.67 (t, *J* = 5.1 Hz, 2H,), 3.95 (t, *J* = 5.1 Hz, 2H), 4.94 (s, 1H, NH), 7.09 (s, 1H), 8.00 (s, 1H), 8.52 (d, J = 2 Hz, 1H), 8.55 (d, *J* = 2 Hz, 1H); anal. calcd for C_15_H_18_N_4_O: C, 66.7; H, 6.7; N, 20.7; found: C, 67.0; H, 7.1; N, 20.2.

## Supporting Information

File 1 Experimental and calculated IR spectra of **3**, UV–vis absorption spectra of **3** in toluene and its fitting with Pekarian function, NMR spectra.
