# Supplementary material for: 1,2,3,4-Tetrahydro-1,4,5,8-tetraazaanthracene revisited: properties and structural evidence of aromaticity loss
Source: Beilstein J Org Chem. 2019 Aug 28;15:2059–68. doi: 10.3762/bjoc.15.203 (PMC6720741; doi:10.3762/bjoc.15.203)
Supplement: File 1 — Experimental and calculated IR spectra of 3, UV–vis absorption spectra of 3 in toluene and its fitting with Pekarian function, NMR spectra. [file Beilstein_J_Org_Chem-15-2059-s001.pdf]

BEILSTEIN JOURNAL OF ORGANIC CHEMISTRY

Supporting Information

for

1,2,3,4-Tetrahydro-1,4,5,8-tetraazaanthracene revisited: properties and structural evidence of aromaticity loss

Arnault Heynderickx, Sébastien Nénon, Olivier Siri, Vladimir Lokshin and Vladimir Khodorkovsky

Beilstein J. Org. Chem. **2019,** *15,* 2059–2068. doi:10.3762/bjoc.15.203

Experimental and calculated IR spectra of 3, UV–vis absorption spectra of 3 in toluene and its fitting with Pekarian function, NMR spectra

License and Terms: This is a supporting information file under the terms of the Creative Commons Attribution License (http://creativecommons.org/licenses/by/4.0). Please note that the reuse, redistribution and reproduction in particular requires that the authors and source are credited. The license is subject to the *Beilstein Journal of Organic Chemistry* terms and conditions: (https://www.beilstein-journals.org/bjoc)

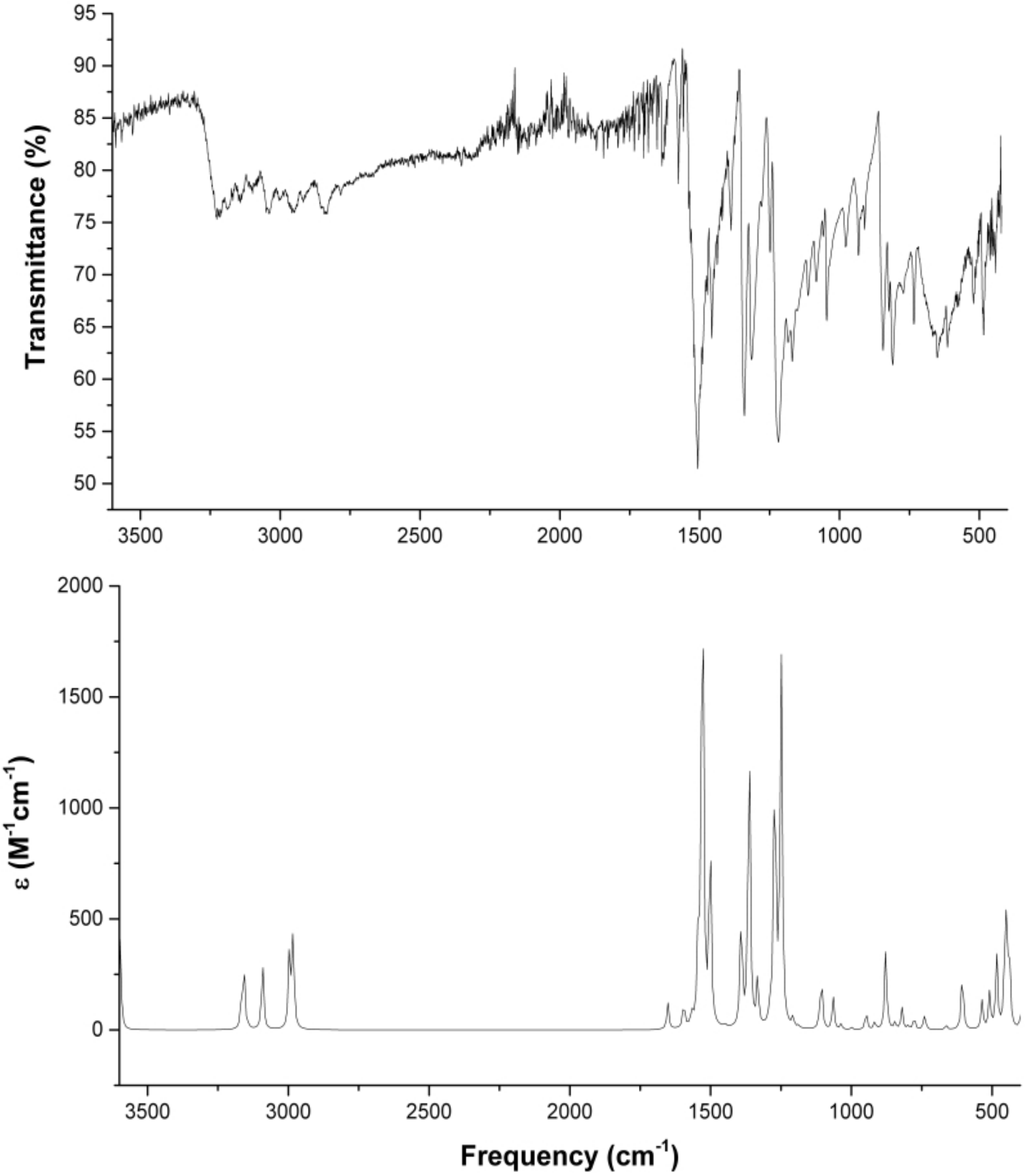

Figure S1. a) IR spectrum of solid sublimed **3**. Calculated by B3LYP/6-311+G(2d,p).

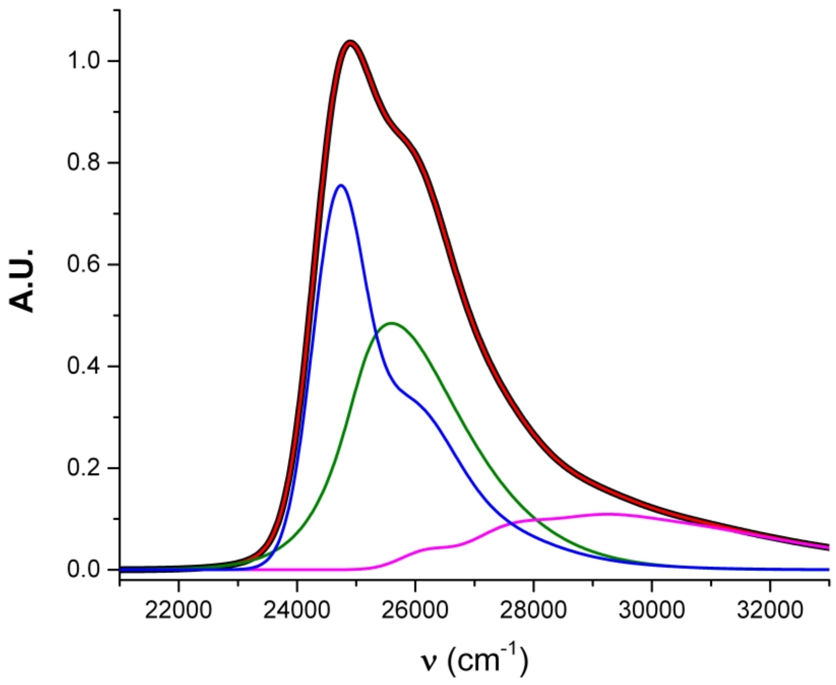

Figure S2. Fitting the experimental UV-Vis absorption spectrum of **3** in toluene at room temperature with Pekarian functions (PF), R^2 = 0.99998. Black: experimental spectrum; blue: PF1, 24671 cm^{-1} (405 nm), 44.3% area; green: PF2, 25197 cm^{-1} (397 nm), 38.4% area; PF3, 26140 cm^{-1} (383 nm), area 17.2%; red: the sum of PF1, PF2 and PF3.

Table S1. TD B3LYP/6-311+G(2d,p) calculated wavelengths (6 states) and oscillator strengths (f) for **3**.

Vacuum			Toluene	
Exc. State	Wavelength λ, nm	f	Wavelength, λ , nm	f
1[a]	351.60	0.24	368.13	0.37
2	339.77	0.003	348.32	0.008
3	337.86	0.006	335.00	0.003
4	281.35	0.006	276.16	0.04
5	262.85	0.03	261.88	0.07
6	258.14	0.06	258.72	0.06

[a] HOMO -> LUMO transition

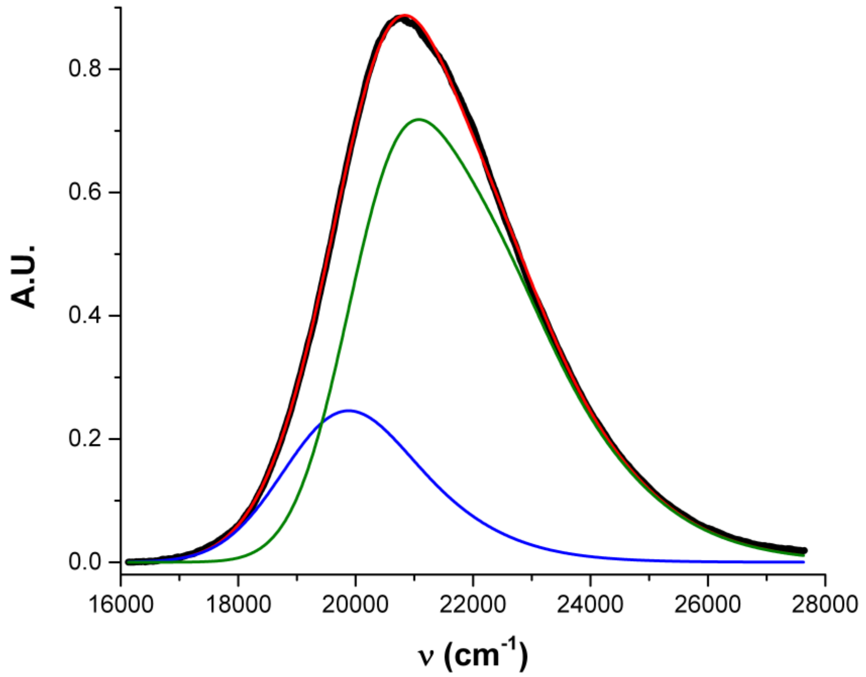

Figure S3. Fitting the experimental UV-Vis absorption spectrum of **6** in water at room temperature with Pekarian functions (PF), R^2 = 0.9998. Black: experimental spectrum; blue: PF1, 19595 cm^{-1} (510 nm), 22% area; green: PF2, 20589 cm^{-1} (486 nm), 78% area; red: the sum of PF1 and PF2.

Table S2. TD B3LYP/6-311+G(2d,p) calculated wavelengths (6 states) and oscillator strengths (f) for **6**.

Vacuum			Ethanol	
Exc. State	Wavelength λ, nm	f	Wavelength, λ , nm	f
1	483.50	0.001	483.49	0.001
2[a]	442.97	0.38	456.05	0.50
3	286.72	0.046	286.85	0.07
4	259.35	0.000	258.02	0.003
5	259.01	0.04	253.54	0.045
6	237.55	0.35	241.06	0.95

[a] HOMO -> LUMO transition

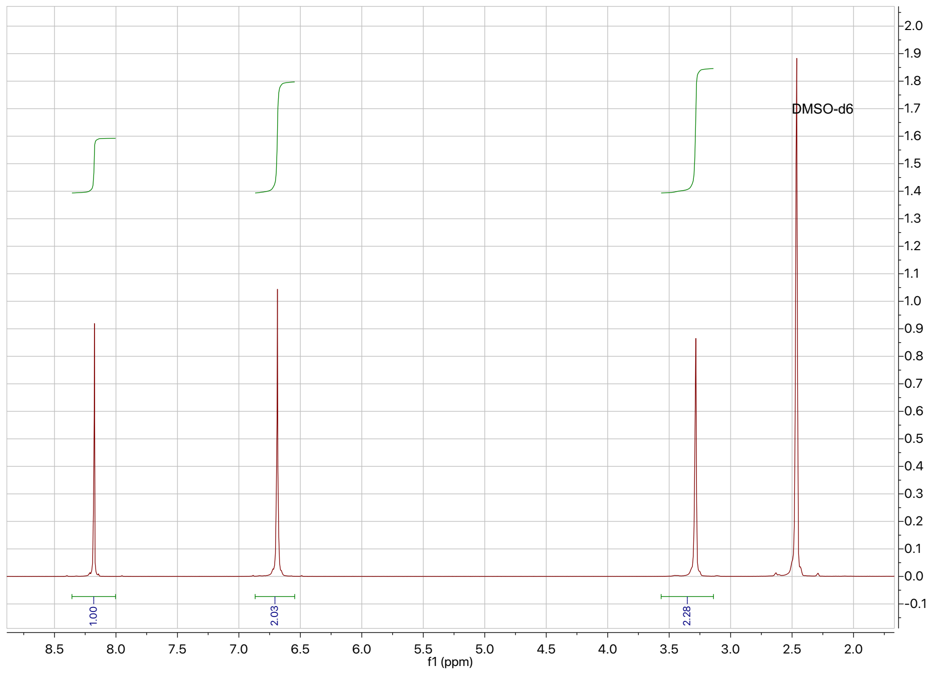

Figure S4. ^{1}H NMR of **3** in DMSO-d_6.

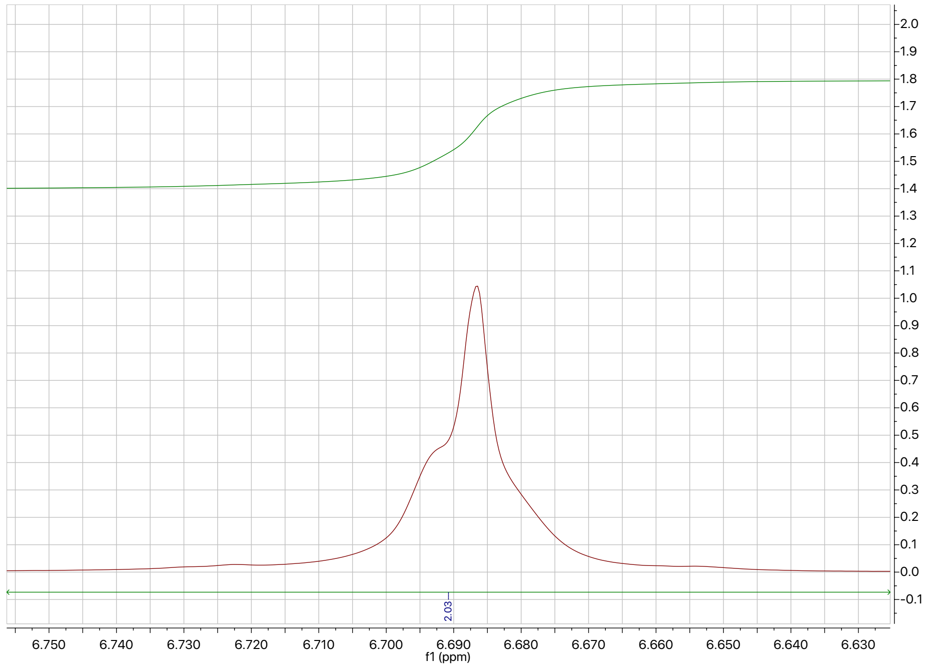

Figure S5. ^{1}H-NMR of **3** in DMSO-d_6, extension.

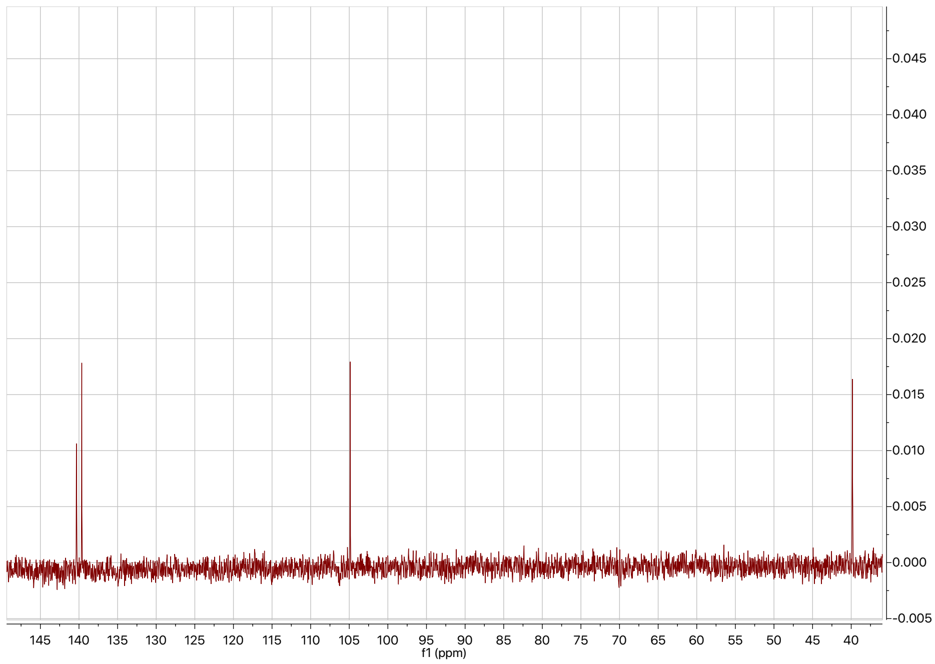

Figure S6. ^{13}C NMR of **3** in DMF-d_7.

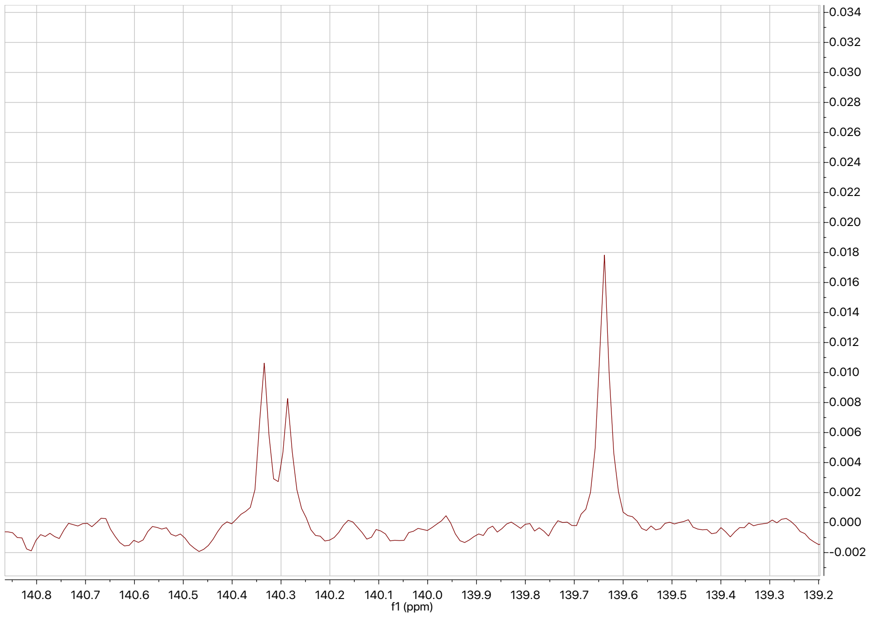

Figure S7. ^{13}C NMR of **3** in DMF-d_7 extension.

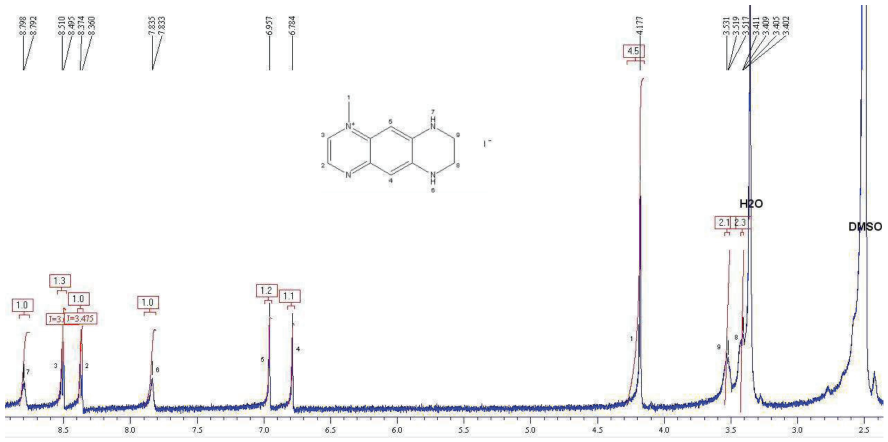

Figure S8. ^{1}H NMR of **7a** in DMSO-d_6.

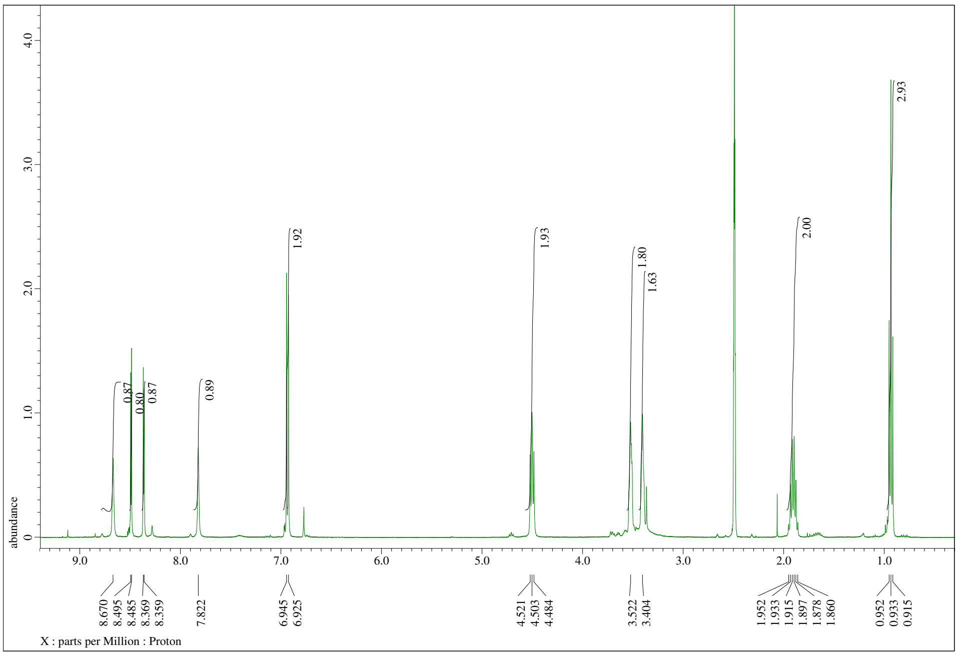

Figure S9. ^{1}H NMR of **7b** in DMSO-d_6.

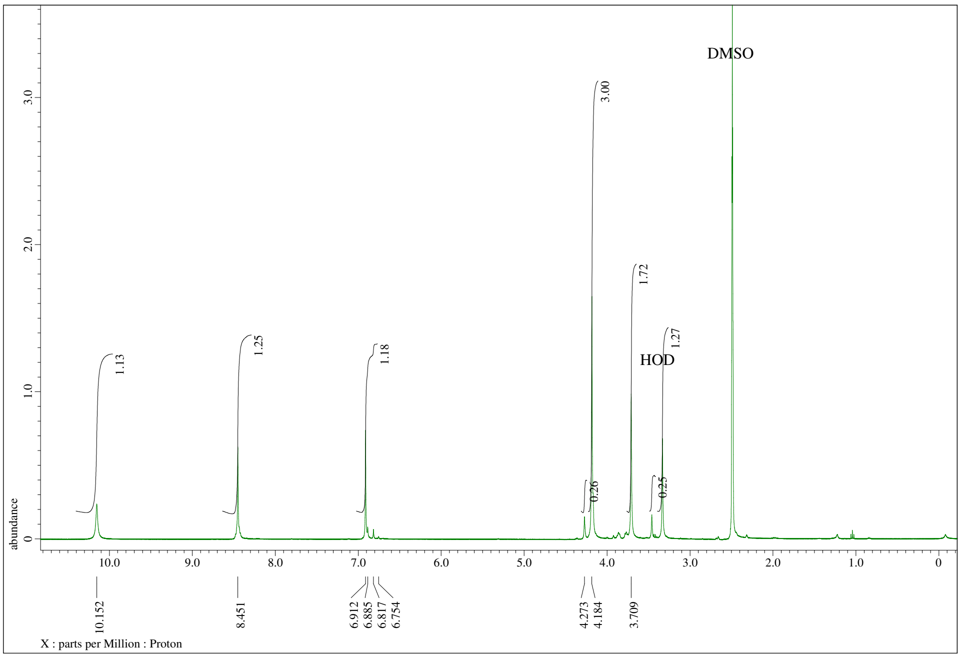

Figure S10. ^{1}H NMR of **8** in DMSO-d_6.

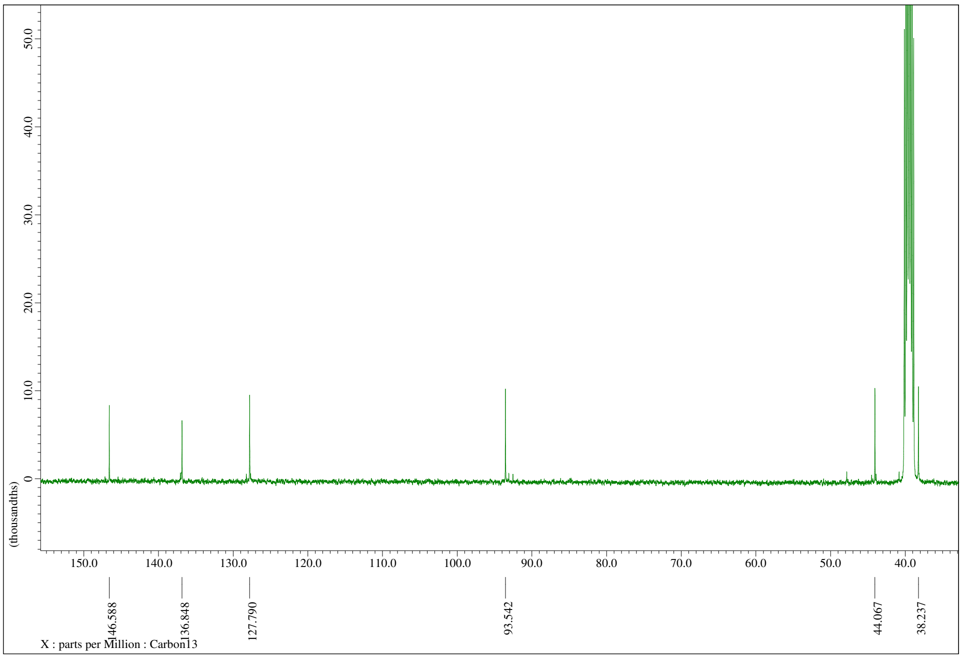

Figure S11. ^{13}C NMR of **8** in DMSO-d_6.

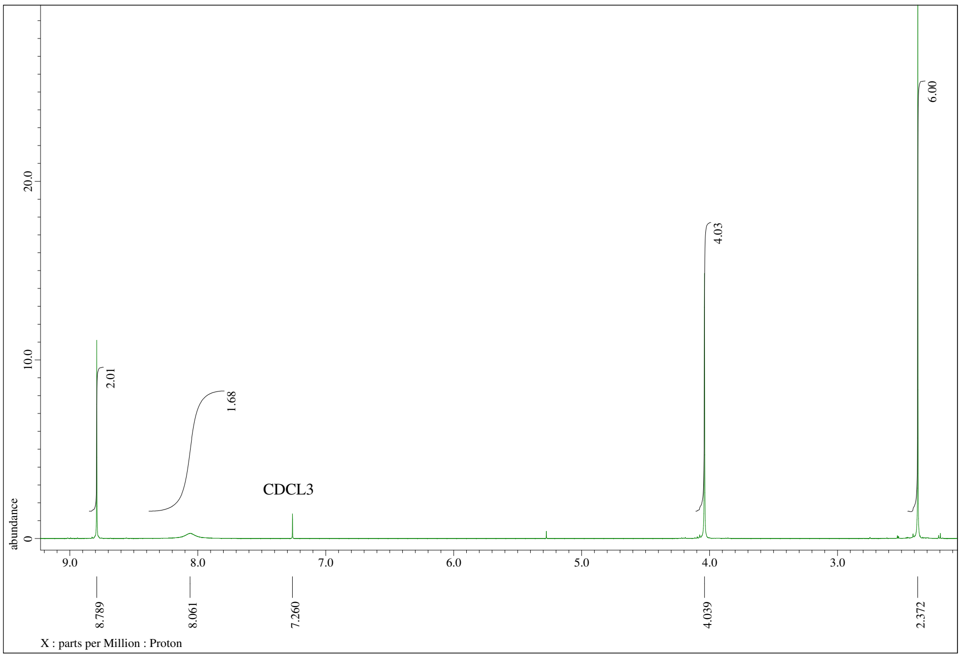

Figure S12. ^{1}H NMR of **9** in $CDCl_3$.

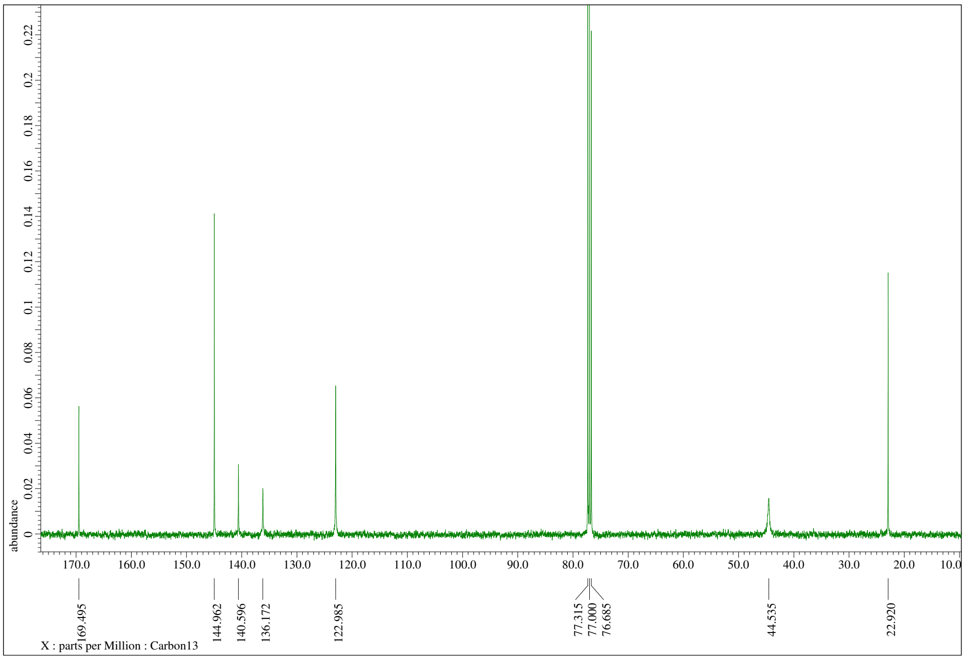

Figure S13. ^{13}C NMR of **9** in $CDCl_3$.

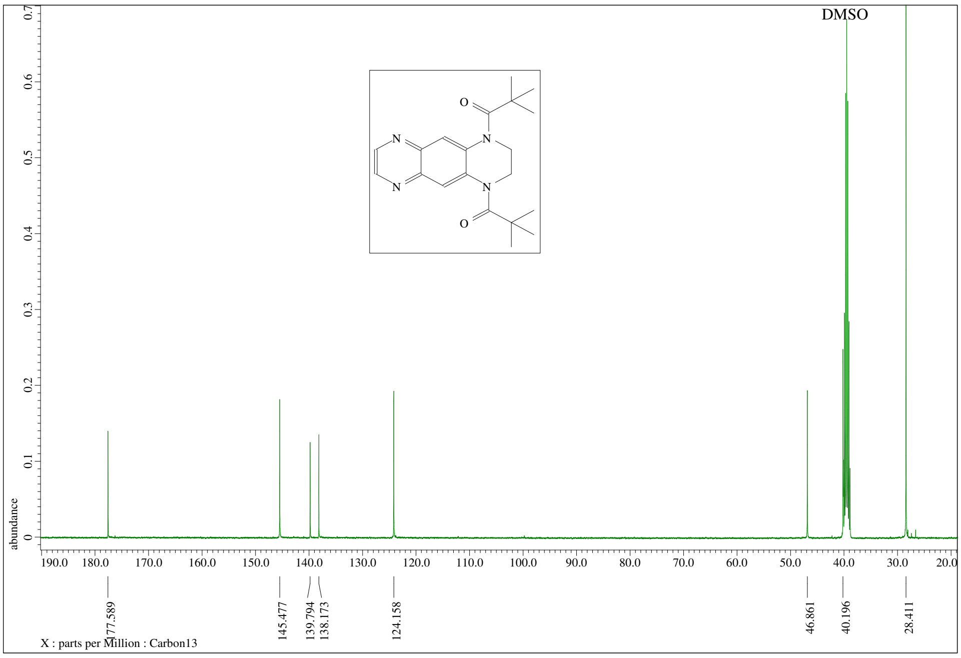

Figure S14. ^{1}H NMR of **10a** in DMSO-d_6.

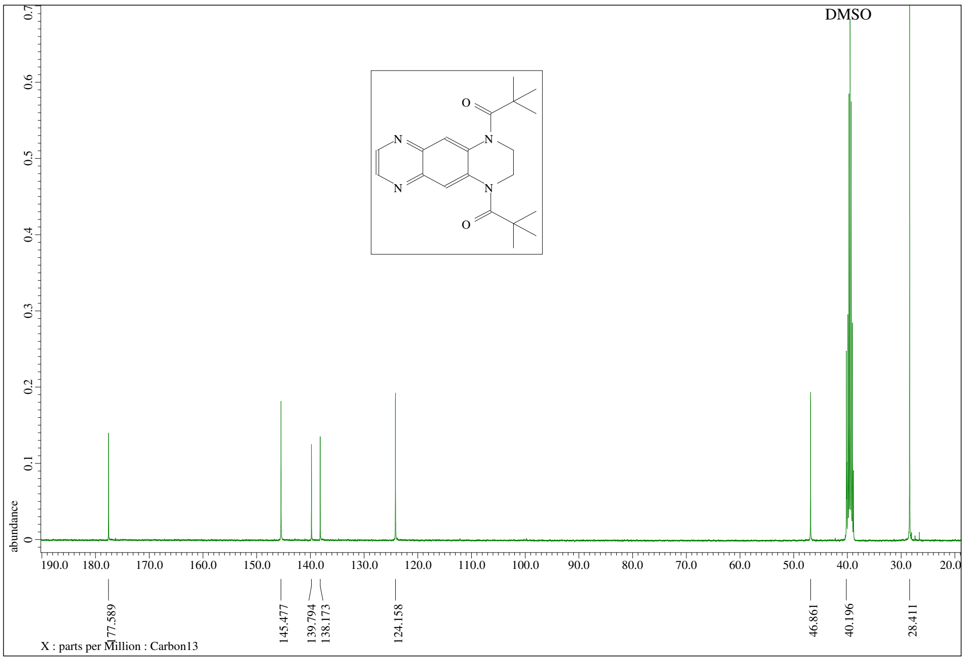

Figure S15. ^{13}C NMR of **10a** in DMSO-d_6.

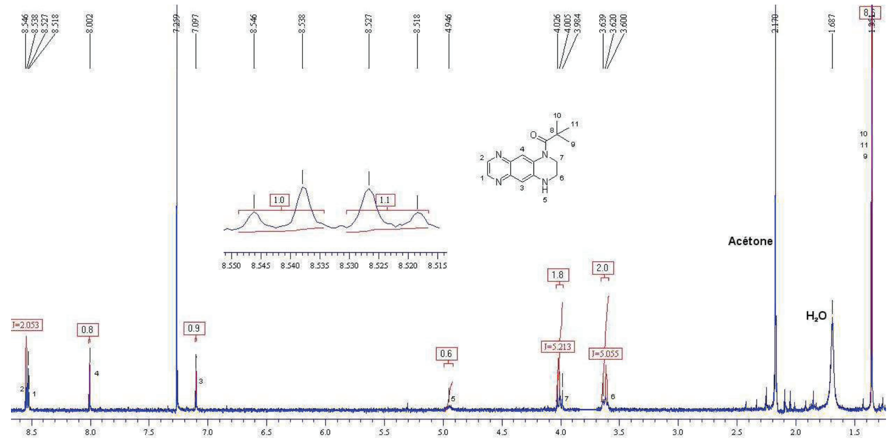

Figure S16. ^{1}H NMR of **10b** in $CDCl_3$.
